# Reconstructive Treatment of Ruptured Intracranial Spontaneous Vertebral Artery Dissection Aneurysms: Long-Term Results and Predictors of Unfavorable Outcomes

**DOI:** 10.1371/journal.pone.0067169

**Published:** 2013-06-26

**Authors:** Kai-Jun Zhao, Yi-Bin Fang, Qing-Hai Huang, Yi Xu, Bo Hong, Qiang Li, Jian-Min Liu, Wen-Yuan Zhao, Ben-Qiang Deng

**Affiliations:** 1 Department of Neurosurgery, Changhai Hospital, Second Military Medical University, Shanghai, China; 2 Department of Neurology, Changhai Hospital, Second Military Medical University, Shanghai, China; Innsbruck Medical University, Austria

## Abstract

**Introduction:**

Few studies focused on predictors of unfavorable outcomes (modified Rankin Scale, 2–6) after reconstructive treatment of the ruptured intracranial spontaneous vertebral artery dissection aneurysms (ris-VADAs), which was evaluated based on 57 reconstructed lesions in this study.

**Methods:**

Results of 57 consecutive patients (M:F = 29∶28; median age, 48 years; range, 27 to 69 years) harboring 57 ris-VADAs, which were treated with coils combined with single stent(n = 32), double overlapping stents (n = 16), and triple overlapping stents (n = 9) between October 2000 to March 2011, were retrospectively reviewed and analyzed.

**Results:**

The available (n = 54) mean durations of angiographic and clinical follow-ups were 27 months (range, 12 to 78) and 62 months (range, 12 to 132), respectively. The involvement of PICA (*p* = 0.004), size of lesions (*p* = 0.000), quantity of stent (*p* = 0.001), and coil type (*p* = 0.002) affected the immediate obliteration grade, which was only risk factor for angiographic recurrences (*p* = 0.031). Although the post-treatment outcomes did not differ between single stent and multiple stents (*p* = 0.434), 5 angiographic recurrences, 1 rebleeding and 1 suspected rebleeding, all occurred in partial obliteration after single-stent-assisted coiling. Progressive thrombosis and in-stent obliteration were not detected on follow-up angiograms. Older age (odds ratio [OR] = 1.090; 95% confidence interval [CI], 1.004–1.184; *p* = 0.040) and unfavorable Hunt-Hess scale (OR = 4.289; 95%CI, 1.232–14.933; *p* = 0.022) were independent predictors of unfavorable outcomes in the reconstructed ris-VADAs.

**Conclusions:**

Immediate obliteration grade was only risk factor for angiographic recurrence after reconstructive treatment. Unfavorable Hunt-Hess grade and older age were independent predictors of unfavorable outcomes in ris-VADAs.

## Introduction

Intracranial spontaneous vertebral artery dissections (VADs) are increasingly diagnosed as the cause of subarachnoid hemorrhage (SAH) or posterior circulation stroke, particularly in young to middle-aged adults [1]–[2]. When vertebral artery trunk occurs aneurysmal dilatation, the dissection aneurysm forms, increasing fatal bleeding risk in future [3]. In the acute stage, surgery has high risks of treatment-related morbidity and mortality [4], thus deconstructive endovascular treatments, which sacrifice parent artery to obliterate aneurysm, were employed for treatment of these ris-VADAs [5]–[9]. Although the majority of ris-VADAs may be treated by either internal coil trapping [5]–[6] or proximal occlusion [7]–[9] of the parent artery, ischemic events or rebleeding still happened in some cases [7] [9]. Besides, the deconstructive treatment was also not suitable for some ris-VADAs, such as hypoplastic or occlusive contralateral vertebral artery and some PICA-involving lesions. Additionally, the deconstructive treatment might result in the occurrence or rupture of contralateral VADA(s) and anterior circulation-related aneurysm(s). Moreover, once the artery stenosis forms in future, a compensatory artery path will lack for the obliteration of vertebral artery.

With the continued advances in stent characteristics [10], stent-supported reconstructive techniques for acute symptomatic dissection aneurysms were increasingly emerging as the promising alternative to deconstructive techniques [11]–[13]. Although sole stent therapy was a promising option for unruptured vertebrobasilar dissection [14], it may not completely prevent rebleeding of lesions [15]. Theoretically, stent(s)-assisted coiling can maintain the patency of parent artery and occlude ruptured sites as much as possible. However, little has been known about long-term results and predictors of unfavorable outcomes of reconstructive treatment for ris-VADAs. In this study, we aimed to evaluate the results of endovascular treatment by using1 to 3 stent(s) with coils based on 57 ris-VADAs with long-term follow-ups.

## Materials and Methods

The Ethics committee of Second Military Medical University affiliated Changhai Hospital approved this retrospective study, and all written consents given by the patients were stored in Changhai hospital database and used for research.

### Patients

A retrospective chart review was conducted for all patients with VADAs who underwent endovascular treatment between October 2000 to March 2011 to identify those patients with ris-VADAs reconstructed with stent(s). All subjects met the following inclusion criteria: (1) history of SAH related to intracranial VADAs, which was confirmed by angiography; (2) initial treatment with stent(s) and coils. Exclusion criteria: (1) traumatic and iatrogenic history; (2) unruptured lesions; (3) lesions extending to extracranial V_3_ portion or basilar artery; (4) initial treatment with deconstructive therapy (n = 25; (5) intracranial bilateral VADAs treated by different methods (n = 10); (6) patients without clinical follow-ups (n = 3). A total of 57 consecutive patients (M:F = 29∶28; median age, 48 years; range, 27 to 69) with 57 ris-VADAs (Hunt-Hess grade: I-II in 43, III in 11, IV in 3, Table 1) were identified and enrolled in this study.

**Table 1:** Comparison of Immediate Angiographic Obliteration Grade.

Variables	Immediate Angiographic Results				
	Complete Obliteration(n = 17)	Partial Obliteration(n = 40)	Total(n = 57)	*P* *Value*	*P, Regression*(OR, 95% CI)
**Age, y, Mean±SD**	51.24±10.51	47.80±10.19		0.263	
**Sex, n (%)**				0.084	0.788 (1.257, 0.237–6.670)
Male	8 (27)	22 (73)	30		
Female	8 (30)	19 (70)	27		
**Hunt&Hess Scale, no. (%)**				0.201	
I–II	9 (21)	34 (79)	43		
III	6 (55)	5 (45)	11		
IV	1 (33)	2 (67)	3		
**PICA Involvement**				**0.004**	0.093 (4.381, 0.781–24.557)
Yes	4 (13)	26 (87)	30		
No	13 (48)	14 (52)	27		
**Aneurysmal Type, no. (%)**				0.073	0.042 (0.114, 0.014–0.926)
Dilatation without stenosis	12 (38)	20 (62)	32		
Dilatation with stenosis	4 (16)	21 (84)	25		
**Blood Supply to Basilar Artery**				0.072	0.746 (1.440, 0.159–13.023)
Predominant	1 (7)	13 (93)	14		
Equal	16 (37)	27 (63)	43		
**Maximum dimension (mm)**				**0.000**	0.102 (4.801, 0.732–31.507)
<10	21 (60)	14 (40)	35		
10–25	2 (9)	20 (91)	22		
**Treatment Interval**				0.217	
≤72 h	9 (24)	28 (76)	37		
>72 h	8 (40)	12 (60)	20		
**Stent (s) Implantation, no. (%)**				**0.001**	0.232 (3.357, 0.460–24.481)
Single stent+coils	4 (13)	28 (87)	32		
Mutiple stents+coils	13 (52)	12 (48)	25		
**Coils, no. (%)**				**0.002**	0.090 (5.868, 0.758–45.436)
Bare Coils	6 (16)	31 (84)	37		
Modified (Hydrocoil/soft) Coils	11 (55)	9 (45)	20		

OR, odds ratio; CI, confidence interval; SD, standard deviation; PICA, the posterior inferior cerebellar artery.

### Aneurysms

VADAs were diagnosed when angiographic imaging revealed fusiform or irregular dilatation with or without stenosis in the affected vertebral trunk portion, then the lesions were subdivided into “dilatation without stenosis” group (fusifum, n = 26; lateral protrusion, n = 7) and “dilatation with stenosis” group (i.e, pearl-and-string, n = 24) based on morphologic presentation. There were 22 (39%) large (10–25 mm) and 35 (61%) small (<10 mm) aneurysms based on the maximum dimension. According to the location of aneurysms relative to the posterior inferior cerebellar artery (PICA), the ris-VADAs were classified as proximal (n = 8), involving (n = 30) and distal (n = 19). Additionally, 14 lesions, including 10 involving PICA lesions, were located at vessels where there was predominant blood supply to basilar artery, 43 located at vessels where there was equal blood supply to basilar artery.

### Procedures

Although all patients were treated immediately after admission for the concern of rebleeding, the mean interval time of between SAH to treatment was 4 days (range, 1–20 days ) because 28 patients were from other hospitals. Regardless of acute phase (≤72 hours) or non-acute phase (>72 hours), the patients with ris-VADAs who underwent a reconstructive treatment were all preloaded rectally 2 hours just before procedure with antiplatelet medication (300 mg aspirin and 300 mg clopidogrel). All patients received systemic intravenous heparin at the beginning of the procedure with activated clotting time between 250 to 300 seconds during procedure. In the early period, the balloon-expandable stent was employed. Later, self-expanding neurovascular stent(s) (Neuroform [Boston Scientific, Natick, Massachusetts], Leo [Balt Extrusion, Montmorency, France], Enterprise [Cordis]) was/were preferred for reconstruction of the entire dissected segment based on the operator’s experience, anatomic characteristics of the affected and contralateral segments, and hemodynamic modification status after deployment of the first stent. Stent placement was defined as a successful implantation if the deployed single stent or overlapped multiple stents in a telescopic fashion extended at least 5 mm over the aneurysm neck (lateral protrusion-shaped dissection aneurysms) or the border of other dissection lesions (i.e. fusiform-shaped, et al) on both sides of the target vessel. After the procedure, with or without 3 days of heparin (40 mg, every 12 hours, hypodermic injection), patients were kept on a dual antiplatelet regimen for 6 weeks with 75 mg Plavix and 100–300 mg aspirin daily, followed by aspirin (100 mg) mono-therapy indefinitely.

### Follow-ups

Angiographic follow-up was performed with conventional angiography, and time intervals were scheduled at 3 to 6 months, then again at 12 months, and annually thereafter. The immediate obliteration grade was defined as complete obliteration if aneurysmal sac was completely obliterated and partial obliteration if aneurysmal sac size remarkably decreased but remained. With reference to the control angiogram performed immediately after treatment, when the contrast medium filled the portion of lesions and revealed no filling, substantial shrink, no change, or increase, angiographic cure, improvement, stability or recurrence were diagnosed, respectively. Clinical outcomes were assessed using the modified Rankin Scale (mRS) score through neurologic examination or telephone interview [16]. The results were categorized into favorable outcomes (mRS, 0–1) and unfavorable outcomes (mRS, 2–6)[17] based on the most recent clinical follow-up at the time of the study.

### Statistical Analysis

Statistical analysis was performed using SPSS for Windows (SPSS17.0). The groups were compared using the Mann-Whitney *U* test for continuous variables, and the *x* ^2^ test and its continuity correction or Fisher exact test for categorical variables, as appropriate. Univariate analysises, including characteristics of patients and aneurysms, were performed to determine the association of angiographic or clinical results with other factors. Then, logistic-regression analysis was performed to determine the independent association of Immediate angiographic results or unfavorable outcomes with other factors. The cutoff in the univariate analysis was *p*<0.20. A *p*<0.05 was considered statistically significant.

## Results

### Treatment and Angiographic Results

All ruptured lesions were reconstructed by using coils and stent(s): single stent (n = 32), double overlapping stents (n = 16), and triple overlapping stents (n = 9), achieving 100% technical success rate. Two treatment-related complications (4%, 2/57) were as follows: one ris-VADA in accompany with focal vasospasm was completely occluded with 3 Enterprises and coils, but the worsening ischemic event resulted in patient’s death 2 days after treatment; the other ris-VADA occurred immediate PICA obliteration after the treatment of 3 stents and coils. Additionally, one rebleeding and one suspected rebleeding patients who did not undergo angiographic follow-ups died 48 hours and 6 months after coils and single stent reconstruction, respectively. Thus, the periprocedural complication rate was 5% (3/57), the suspected late complication rate was 2% (1/57), and the overall mortality rate was 5% (3/57).

**Figure 1:**
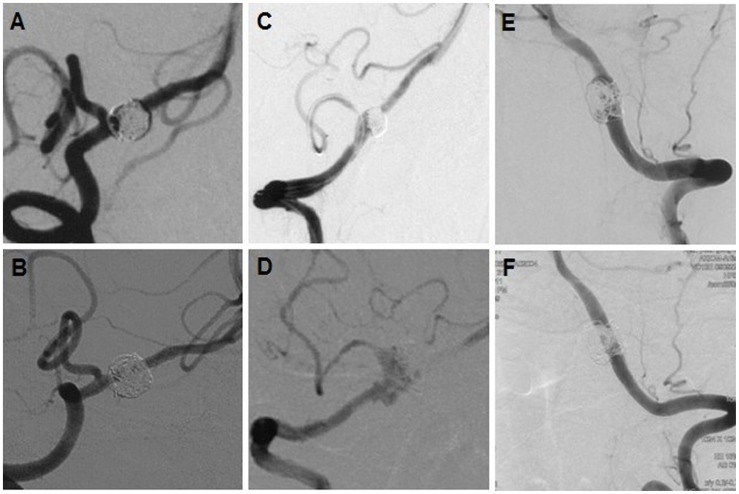
Reconstructive treatment of ris-VADAs. (A–B) a ris-VADA was treated with single balloon-expandable stent, showing partial obliteration (A), 78 months follow-up imaging (B) confirmed complete obliteration. (C–D) a ris-VADA with partial obliteration (C) recurred (D) 3 months after treatment of single stent. (E–F) a ris-VADAs with partial obliteration was cured 12 months after treatment using 3 Enterprise stents.

The immediate obliteration grades were summarized in Table 1–2. A higher rate of partial obliteration (Table 1) was observed in the PICA-involving group (87% vs 52% in the PICA non-involving group, *p* = 0.004), in maximum dimension(10–25 mm) group (91% vs 40% in maximum dimension (10 mm) group, *p* = 0.000), in the single-stent group (87% vs 48% in the multiple-stent group, *p* = 0.001), and in the bare-coils group (84% vs 45% in the modified-coil [hydrocoil/hydrosoft] group, *p* = 0.002). However, the forgoing factors were all not independent predictors in logistic regression analysis (Table 1). The angiographic follow-ups (Table 2) of the surviving 54 patients were available for a mean of 27 months (range, 12 to 78 months), showing complete obliteration in 42 lesions, improvement or stability in 7, and recurrence in 5. Moreover, the immediate partial obliteration after initial treatment was the only risk factor for angiographic recurrence (*p* = 0.031, Table 2) on univariate analysis. Of those with complete obliteration (n = 30) after initial treatment, none developed recurrence (*p* = 0.031). Although the treatment of ris-VADAs with coiling and single stent was effective (Fig. 1A–1B), all 5 recurrences had 2 the common risk profile: (1) therapy with single stent and coils; (2) partial obliteration (Fig. 1C). Progressive thrombosis, in-stent stenosis, and in-stent obliteration were not detected on follow-up angiograms.

**Table 2:** Risk factors for Angiographic Recurrence after Endovascular Treatment in ris-VADAs (n = 54).

Variables	Cure, Improvement,and Stability (n = 49)	Recurrence(n = 5)	Total	*p*Value
**Age, y, Mean±SD**	49.51±10.29	40.20±9.26		0.080
**Sex, n (%)**				0.348
Male	23 (85)	4 (15)	*27*	
Female	26 (96)	1 (4)	27	
**Hunt&Hess Scale, no. (%)**				0.440
I–II	36 (88)	5 (12)	41	
III–IV	13 (100)	0 (0)	13	
**PICA Involvement**				0.394
Yes	24 (86)	4 (14)	28	
No	25 (96)	1 (4)	26	
**Aneurysmal Type, no. (%)**				0.550
Dilatation without stenosis	27 (87)	4 (13)	31	
Dilatation with stenosis	22 (96)	1 (4)	23	
**Blood Supply to Basilar Artery**				1.000
Predominant	11 (92)	1 (8)	12	
Equal	37 (88)	5 (12)	42	
**Treatment Interval**				0.245
≤72 h	31 (86)	5 (14)	36	
>72 h	18 (100)	0 (0)	18	
**Stent(s) Implantation, no. (%)**				0.104
Single stent+coils	25 (83)	5 (17)	30	
Multiple stents+coils	24 (100)	0 (0)	24	
**Maximum dimension (mm)**				
<10	31 (94)	2 (6)	33	0.300
10–25	17 (81)	4 (19)	21	
**Coils, no. (%)**				0.216
Bare Coils	30 (86)	5 (14)	35	
Modified (Hydrocoil/soft) Coils	19 (100)	0 (0)	19	
**Immediate Obliteration Grade, no. (%)**				**0.031**
Complete obliteration	30 (100)	0 (0)	30	
Partial obliteration	19 (79)	5 (21)	24	

ris-VADAs, the ruptured intracranial spontaneous vertebral artery dissection aneurysms; PICA, the posterior inferior cerebellar artery.

### Clinical Follow-ups

Clinical follow-ups of the surviving 54 patients were available for a mean of 62 months (range, 12 to 132 months). One rebleeding and one suspected rebleeding patients who did not undergo angiographic follow-ups died 48 hours and 6 months after treatment, respectively. Of 57 patients with ris-VADAs, 47 had favorable outcomes (mRS, 0–1), which was more frequently observed in Hunt-Hess grade I–II group (91% [39/43]), followed by Hunt-Hess grade III group (73% [8/11], Table 3). All 3 patients with Hunt-Hess IV had unfavorable outcomes (mRS, 2–6). Furthermore, favorable outcomes were more frequently observed in younger patients (83%, 47.11±9.44 years, *p* = 0.021, Table 3) as well. Older age (OR = 1.090; 95% CI, 1.004 to 1.184; *p* = 0.040) and unfavorable Hunt-Hess scale (OR = 4.289, 95% CI, 1.232 to 14.933; *p* = 0.022) were independent predictors of unfavorable outcomes in the logistic regression analysis (Table 3).

**Table 3:** Predictors of Unfavorable Outcomes in ris-VADAs.

Variables	Favorable Outcomes(n = 47)	Unfavorable Outcomes(n = 10)	Total(n = 57)	*p*Value	*P, Regression*(OR, 95% CI*)*
**Age, y, Mean±SD**	47.11±9.44	56.90±10.88		**0.021**	0.040 (1.090, 1.004–1.184)
**Sex, n (%)**				1.000	
Male	24 (83)	5 (17)	29		
Female	23 (82)	5 (18)	28		
**Hunt&Hess Scale, no. (%)**				**0.000**	0.022 (4.289, 1.232–14.933)
I–II	39 (91)	4 (9)	43		
III	8 (73)	3 (27)	11		
IV	0 (0)	3 (100)	3		
**PICA Involvement**				0.388	
Yes	23 (77)	7 (23)	30		
No	24 (89)	3 (11)	27		
**Aneurysmal Type, no. (%)**				0.616	
Dilatation without stenosis	26 (79)	7 (21)	33		
Dilatation with stenosis	21 (88)	3 (12)	24		
**Blood Supply to Basilar Artery**				1.000	
Predominant	12 (86)	2 (14)	14		
Equal	35 (81)	8 (19)	43		
**Maximum dimension (mm)**				0.387	
<10	30 (86)	5 (14)	35		
10–25	16 (73)	6 (27)	22		
**Treatment Interval**				1.000	
≤72 h	31 (84)	6 (16)	37		
>72 h	16 (80)	4 (20)	20		
**Stent(s) Implantation, no. (%)**				0.434	
Single stent+coils	28 (88)	4 (12)	32		
Multiple stents+coils	19 (76)	6 (24)	25		
**Coils, no. (%)**				0.069	0.210 (2.899, 0.550–15.294)
Bare Coils	33 (89)	4 (11)	37		
Modified(Hydrocoil/soft) Coils	14 (70)	6 (30)	20		
**Obliteration Grade, no. (%)**				0.694	
Complete obliteration	13 (76)	4 (24)	17		
Partial obliteration	34 (85)	6 (15)	40		

OR, odds ratio; CI, confidence interval; ris-VADAs, the ruptured intracranial spontaneous vertebral artery dissection aneurysms; OR, odds ratio; CI, confidence interval; PICA, the posterior inferior cerebellar artery.

## Discussion

**Figure 2:**
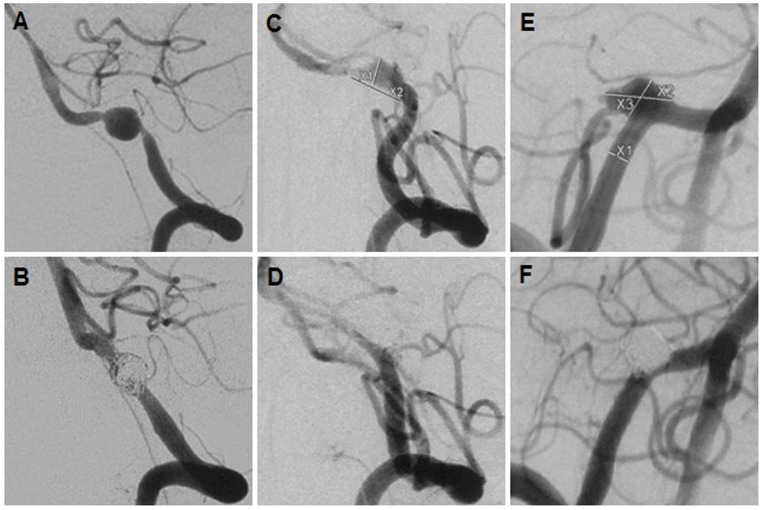
Three adverse events. A ris-VADA (A) was treated by single stent and coiling,(B) but rebleed 48 hours after treatment; A ris-VADA in accompany with focal vasospasm (C) was completely occluded (D) with 3 Enterprises, but the worsening ischemic event resulted in death; A ris-VADA (E) was completely occluded with 3 Enterprises, showing immediate PICA obliteration (F).

Rupture and rebleeding of VADAs were closely related to their angiographic configurations [18]–[19]. This series of all ruptured aneurysms all had dilated configurations (100%, Table1), which was in favor of the view that dilated lesions (dilatation without stenosis or pearl-and-string) were more likely to rupture [19]. In contrast, the stenosis and dilatation configurations might be prone to rebleeding after treatment, particularly when involving PICA, such as the lesion in Fig. 2A–2B. As was well favored by another study where angiographic configurations such as “stenosis and dilation” or “location at or proximal to the PICA origin” were two independent predictions of rebleeding after treatment [18]. One possible explanation was that the configurations of dilation with stenosis may have more fragile wall.

Although the complete obliteration remained the primary goal of endovascular coiling of intracranial aneurysms [20], the immediate complete obliteration of large lesions remains challenging (*p* = 0.000,Table 1). Our data and literature indicated modified coils were helpful to increase the immediate obliteration grade (*p* = 0.002, Table1) and decrease the recurrence rate [21]. However, when PICA-involving lesions were reconstructed, aneurysmal sac had to be left partially open to ensure flow through it to PICA. Compared with its competitor-PICA non-involving lesions, a higher rate of partial obliteration (87%) was observed in PICA-involving group (*p* = 0.004, Table1), increasing recurrent risks (Table 2, 14% vs 4%), which was in favor of the viewpoint that PICA origin involvement was a risk factor for post-procedural recurrence [11]. However, that study did not perform the association between recurrence and immediate obliteration grade. In consideration of recurrence and rebleeding after treatment, descontructive treatment might be selected for these ris-VADAs for the immediate complete obliteration. However, ischemic events may be encountered, especially in lesions involving PICA or lesions located on the side with predominant blood supply to basilar artery or both, such as this series of cases (PICA-involving, n = 30; predominant flow supply, n = 14; both, n = 10). It was of particular note that multiple stents in comparison with single stent evidently enhanced immediate aneurysmal obliteration grade (Table1, *p* = 0.001) which was closely related to angiographic recurrences (Table 2, *p* = 0.031). Moreover, favorable angiographic results (cure, improvement, or stablility) were more frequently observed in the multiple-stent group (24/24, 100%) than in single-stent group (25/30, 83%). Five angiographic recurrences (ie, Fig. 1D), 1 rebleeding (Fig. 2A–2B) and 1 suspected rebleeding commonly underwent single-stent-assisted coiling, and resulted in initial partial obliterations. This indicated that partial obliteration following single-stent-assisted coiling had higher risk of recurrence and rebleeding. In contrast, none of ris-VADAs in the multiple-stent group had rebleeding or angiographic recurrence during long-term follow-ups, even in the cases of partial obliteration (ie.Fig. 1E–1F). These findings suggested that multiple overlapping stents might be more effective in preventing angiographic recurrence and rebleeding through the increasement of immediate obliteration grade (Table 1). Several experimental studies were in favor of these results. Firstly, stent-related favorable hemodynamic modifications were helpful to prevent coil compaction, aneurysmal rupture and growth [22]. Secondly, these favorable hemodynamic changes were in proportion to the number of overlapping stents [23]–[24]. The strengthes of the overlapping stents may be attributing to low stent porosity and the effective straightening curvature of parent vessel. Currently, the multiple stents technique was preferred at our center for the treatment of ruptured or unruptured VADAs under the following circumstances: (1) the lesion with partial obliteration after single stent implantation with or without coiling; (2) The PICA-involving lesions, especially in the circumstances that the contralateral PICA is absent and the ipsilateral anterior inferior cerebellar artery is hypoplastic; (3) the VADA is also located at contravertebral artery; (4) contralateral vertebral artery is hypoplastic or occlusive.

In the early period, one confirmed rebleeding and one suspected rebleeding patient underwent single stent and coils treatment and both subsequently died. Preventing fatal rebleeding [4], [6], [8], [19], might be more important than ensuring the patency of PICA. In the later period, with increasing clinical experience and experimental knowledges, multiple overlapping stents combined with coils were preferred for treatment of some ris-VADAs, especially in PICA-involving lesions, to address the concern of rebleeding. Meanwhile, our post-procedural medicine protocol was modified as well. Three days of heparin was no longer used, and we substituted 100 mg aspirin and 75 mg clopidogrel for 300 mg aspirin and 75 mg clopidogrel in the first 6 weeks after procedure. 100 mg aspirin application remained indefinitely. Occasionally, some patients developed obstructive hydrocephalus, and the external ventricular drainage could be firstly performed, followed by reconstructive treatment. In the follow-ups, there were no episodes of rebleeding and the increasement of hemorrhage rates, indicating that our medicine protocol modification was appropriate. However, of those (n = 9) treated with triple stents, 1 ischemic events (Fig. 2C–2D) and 1 immediate PICA obliteration (Fig. 2E–2F) were encountered. Because the porosity of the available three overlapping stent(s) remains lower than flow diverter, the possible explanation involved serious cerebral vasospasm (ie. Fig. 2C–2D), coil migration after stent(s) implantation, or both. Ultimately, the patient with PICA obliteration was discharged home with a mRS score of 4. This was in favor of the view that PICA obliteration might be more tolerable than expected [9], [11], [25].

A study advocated very early angiographic follow-up for PICA-involving ris-VADAs, based on the observation that rebleeding events happened more frequently within 4 days after initial treatment [11]. In that study, all rebleeding events occurred in patients who underwent single-stent-assisted coiling or stent-only implantation [11]. In this series, the PICA-involving lesions were preferably treated with multiple stents and coils, achieving the encouraging results that none rebleeding or recurrence occurred in multiple-stent group during long-term follow-ups. Hence, provided that lesions were treated by multiple stents and coils, the earlier angiographic follow-up might not to be necessary. Another study found, even if ris-VADAs were treated with proximal obliteration, post-procedure angiography remained of only limited value in assessing the possibility of rebleeding [7].

This series of ris-VADAs predominantly occurred in middle-aged adults (median age, 48 years; range, 27 to 69 years), followed by young adults, which was in favor of previous reports [17]–[18], [26]. A recent study confirmed that older age (median age, 46 years) was an independent predictor of unfavorable outcome (mRS, 2–6) in symptomatic intracranial unruptured vertebrobasilar dissection treated with either endovascular or medical therapy [17]. In this series, older age (mean age, 56.90 years; OR = 1.090; 95% CI, 1.004–1.184; *p* = 0.040, Table 3) remained an independent predictor of unfavorable outcomes (mRS, 2–6) in ruptured VADAs. There were several possible explanations for unfavorable outcomes of unruptured or ruptured intracranial spontaneous VADs in the older age group:(1) symptomatic intracranial VADs occurred more frequently with older age and had lesser tendency of spontaneous cure; (2) older patients may have poorer tolerance to SAH; (3) older patients may have longer chronic disease course or different etiologies.

Of the total 57 patients, 47 (83%) had favorable outcomes. 39 (91%) of the 43 patients with Hunt-Hess grade I-II, and 8 (73%) of the 11 patients with Hunt-Hess grade III all showed favorable outcome in the follow-ups. In contrast, outcomes of 3 patients with Hunt-Hess IV were all unfavorable. The logistic-regression analysis indicated the unfavorable Hunt-Hess scale (OR = 4.289; 95%CI, 1.232–14.933; *p* = 0.022) was also an independent predictor of unfavorable outcomes in ris-VADAs after treatment. This was also well favored in a previous long-term follow-up study, which indicated that low pre-procedure Hunt-Hess grade predicted favorable clinical outcomes in ris-VADAs after endovascular treatment [27]. However, another study found that the Hunt-Hess grade in saccular aneurysms did not affect patients’ clinical outcomes [28], which seemed to differ between ruptured saccular and dissection aneurysms. This contradiction was due to the different definition of unfavorable outcomes in that study, where a Glasgow Outcome Scales score of 5 was defined as “good outcome”, a score of “3 or 4” was defined as moderate outcome, and “1 or 2” as poor outcome.

### Limitations

In this series, there are some limitations, such as the retrospective design, patient selection bias, limited cases in a single institution.

### Conclusions

The long-term follow-ups in our patients confirmed the efficacy of reconstructive treatment and the encouraging results of multiple stents in preventing recurrence and rebleeding in ris-VADAs after reconstructive treatment. Unfavorable Hunt-Hess grade and older age were independent risk factors of unfavorable outcomes in ris-VADAs.
